# Variability of Properties of Wood Biomass Combustion Waste During the Heating Season in the Context of Their Environmental Use

**DOI:** 10.3390/ma19071295

**Published:** 2026-03-25

**Authors:** Elżbieta Rolka, Anna Skorwider-Namiotko, Radosław Szostek

**Affiliations:** Department of Agricultural and Environmental Chemistry, Faculty of Agriculture and Forestry, University of Warmia and Mazury in Olsztyn, Łódzki 4 Sq., 10-727 Olsztyn, Poland; anna.namiotko@uwm.edu.pl (A.S.-N.); radoslaw.szostek@uwm.edu.pl (R.S.)

**Keywords:** biomass bioheating plant, wood chips, heating season, combustion waste, pH, salinity, chemical composition, macronutrients, heavy metals

## Abstract

The use of wood chips in the heating sector leads to the generation of combustion waste with variable properties, which poses challenges for their rational management. To determine the variability of combustion waste, samples were collected over a 13-week period during the heating season, as weekly aggregate samples from a biomass bioheating plant burning wood chips. Three waste fractions were obtained for analysis: residue from the grate (B1), dust from the dust collector (B2), and boiler dust (B3). Dry matter (DM), reaction (pH_KCl_), electrolytic conductivity (EC), content of total carbon (TC), total nitrogen (TN), macronutrients (P, K, Mg, Ca, Na), and heavy metals (Fe, Mn, Zn, Cu, Pb, Cd, Cr, Co, Ni) were determined in the collected samples. All waste fractions were characterized by an alkaline reaction. Regardless of the waste fraction, the macronutrient content was dominated by Ca, K, and Mg, with significantly lower levels of P and Na. Among heavy metals, Fe, Mn, and Zn had the highest recorded contents, and the lowest by far was Cd. With respect to sampling dates, the least diversified chemical composition was observed for B1 samples, more diversified for B2, and the most diversified for B3. In turn, regardless of the waste fraction, the most diversified results were observed for Cd and Pb, and the least for pH, DM, and TC. Concerning environmental management of combustion waste, fraction B1 deserves attention, as it was characterized by the richest chemical composition (TN, P, K, Mg, Ca, Na, Mn, Zn, Cu, Co, Ni). However, due to the highest content of undesirable heavy metals (Pb, Cd) and the highest salinity, it requires constant monitoring of the composition.

## 1. Introduction

Many countries are currently seeking new, renewable heat and energy sources with lower emissions. Generating energy from renewable sources is more environmentally friendly, reduces emissions of harmful substances, including greenhouse gases [1], and limits the carbon footprint [2]. The most common renewable energy sources (RESs) for electricity and heat include biomass [3,4,5,6], which is CO_2_-neutral [6], and the use of which is constantly growing [7]. Compared to solar or wind energy, energy produced from biomass is a more stable source, which increases its importance for the development of RESs [1]. Currently, forest wood biomass is a key renewable energy source in the energy transition and climate change mitigation [8]. In the energy sector, wood biomass is most frequently used [1,9] in the form of wood chips, sawdust, pellets, or briquettes [1]. Since wood is a widely available raw material on the market [10], it has become a viable alternative to coal [11]. This is particularly noticeable during the heating season, when low temperatures make it necessary to heat residential buildings and other public buildings. In Poland, coniferous wood dominates wood biomass due to its large share (68%) in wood resources [10]. However, combustion of wood biomass leads to the production of combustion waste [2,11,12]. Burning wood produces 0.4 to 3.9% ash relative to the dry mass of the fuel [6], which depends on the species and part of the wood [13]. Some estimates indicate that biomass combustion can produce approximately 476 million tons of combustion waste annually [6]. However, the amount of ash may vary depending on many factors, including combustion temperature, wood species [13,14], and technology used [14]. In the coming years, an increase in the amount of this waste can be expected since many countries, including Poland, have set an energy target—the development of RESs [4]. Poland is to achieve at least a 23% share of RESs in gross final energy consumption by 2030 [15]. It is estimated that by 2050, 33–50% of global energy resources could be covered by biomass combustion [6]. Therefore, combustion waste generated by biomass combustion should be rationally managed [5,16]; so far, it constitutes a serious environmental and economic problem [11]. The problem with waste management is related to the high variability of chemical composition, which depends on the type of biomass [6,14,17,18], the species and part of the plant [19], the age of the plant [17], growth conditions [19], the species of wood and its storage time [20], processing methods [19], combustion technology and conditions [6,14,17,21], and other technical conditions [19]. The above-mentioned factors may differentiate the content of elements in ash [19]. This heterogeneity of ashes complicates the development of standardized methods of disposal or management [17,22].

For several years, research has been underway on the various uses of wood ash. Attempts have been made to use this material, among other uses, for the synthesis of geopolymers [23], as a filler in asphalt pavements [24], in advanced engineering materials, in the synthesis of biofuels [17], and in construction [11,12,14,25,26,27]. Wood ash is considered in the production of bricks [14] and eco-binders (alkaline-activated materials) [12,28], the creation of ash–cement composites [11,29], and improving the properties of concrete [30]. Such use of ash is possible provided that the material is properly stored [25] and the content of contaminating metals is controlled [31]. To prevent prehydration and carbonization of ash, this material should be stored in closed containers [25], which can be troublesome and significantly limit its use in these applications. Another factor inhibiting the use of wood ash in construction is the high carbon, which reduces the mechanical properties of the produced eco-binders [12]. Therefore, further research is needed in the construction sector on the effect of biomass ash on mechanical properties, durability, and compliance with technical standards when it is to be used as a cement substitute or as an additive to concrete. In the case of catalysis in the synthesis of biofuels, research on the possibility of extracting the desired elements is necessary, as well as on the activity of biomass ash as a catalyst, including its stability and the impact of contaminants [17].

Biomass ash is now increasingly perceived as a secondary source of nutrients in agriculture, which is consistent with the assumptions of a circular economy [32]. Due to its high content of alkaline compounds, wood ash is a good material for liming [3,33,34], especially on medium and heavy soils [35]. This material can be an alternative to commercial agricultural lime in neutralizing the acidic reaction of agricultural soils [34]. Soil application of wood ash allows the introduction of significant amounts of C, N, P, K, Ca, Mg and S [35], increasing the content of exchangeable Ca [36] and phytoavailable forms of P, K, and Mg [37,38,39], as well as the soil sorption complex and its saturation with base cations [38,39]. Due to its high nutrient content, this material improves plant growth [40,41,42], significantly increasing yields [34,42,43,44] and resistance to mechanical damage [44]. The beneficial effects of ash use can be maximized in combination with other organic and inorganic additives [40], e.g., together with digestate [45] or sewage sludge [46,47]. Using wood ash in appropriate proportions with biochar can replace traditional mineral fertilizers and optimize the cultivation of energy crops [37,43] or the production of biomass for feed purposes [47]. To use ash alone more effectively, granulation of this material is being tested in Sweden and Denmark, which is supposed to ensure easier processing, transport, and end use [24]. The possibility of reusing wood ash may become, on the one hand, an opportunity to recover valuable nutrients, and on the other hand, an alternative to storing it in landfills, thus preventing secondary environmental pollution [47]. Due to the significant content of macronutrients [6,40], especially Ca [6,24] and K, as well as microelements [6], ash can be considered a valuable fertilizer component. However, it can also be a source of heavy metals [3,6], especially Cd, Cr, and Hg [24]; polycyclic aromatic hydrocarbons (PAHs); and even volatile organic compounds (VOCs) [6]. Soil application of ash with excessively high Cd and Pb content can lead to the accumulation of these elements in the soil [48]. PAHs and VOCs can be formed during the incomplete combustion of wood in low-power boilers under unstable operating conditions [6]. The source of heavy metals in combustion waste is most often biomass itself. In the case of Ni and Cr, the source may be high-grade steel used in process lines [49], which corrodes due to the corrosive action of biomass during combustion processes [50]. Therefore, according to some researchers [51], the composition of ash changes even when using a single type of biomass. Due to this large variation in ash quality, it is necessary to analyze the chemical composition before determining the direction of its utilization, especially as a fertilizer [19].

The available literature extensively characterizes the composition of ash obtained from different types of biomass or from different bioheating plants [19,31,34,35,52,53], but there is still a lack of information on the chemical composition of ash from a single bioheating plant and its variability over a specific time period. Furthermore, in such plants, dust from baghouse dust collectors is most often combined with ash from the grate, which can lead to excessive accumulation of heavy metals in the waste. To fill this gap, the presented research focused on a single bioheating plant from which combustion waste was obtained during the heating season, and among these, three fractions were identified (B1—residue from the grate; B2—dust from the dust collector; and B3—boiler dust). Therefore, the presented research proposes a new approach to this issue. The research hypothesis assumed that (1) combustion of woody biomass from nearby local sources should not affect the high variability of the chemical composition of combustion waste from a single selected bioheating plant, and (2) combining dust from the dust collector with grate ash may result in excessive accumulation of undesirable components. The aim of this study was to assess the chemical composition of combustion waste fractions from bioheating plants (B1, B2, and B3) selected for environmental testing and to verify the hypothesis’s assumptions.

## 2. Materials and Methods

### 2.1. Research Material

**Figure 1:**
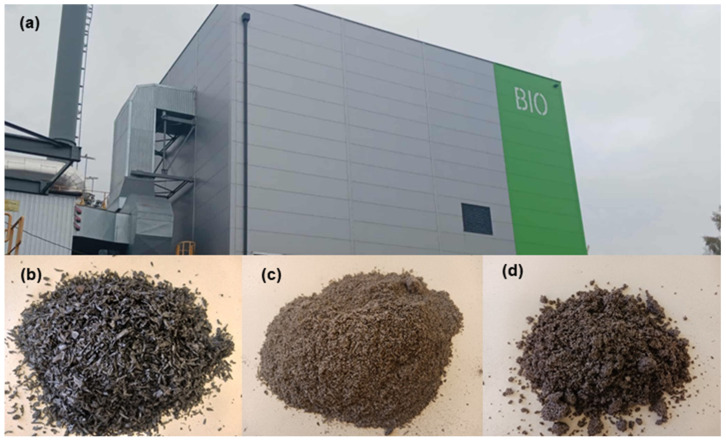
Waste obtained after burning wood biomass: (**a**) Bioheating plant building; (**b**) B1—residue from the grate; (**c**) B2—dust from the dust collector; (**d**) B3—dust from the boiler.

The research material consisted of waste samples obtained from a biomass bioheating plant (Kortowo-BIO) (Figure 1a) from the Municipal Heating Energy Company in Olsztyn (MHEC, Olsztyn, Poland). The Kortowo-BIO bioheating plant has been operating since 2019 and was established as a result of the project entitled “Increasing energy production from renewable sources at MHEC LLC in Olsztyn through the construction of a biomass-based installation.” The facility is equipped with one water boiler with a rated power of 25 MW. The temperature in the combustion chamber is 800–900 °C. Kortowo-BIO is a modern facility equipped with a flue gas heat recovery system (UOC—recovers an additional 5 MW of power), a system reducing nitrogen oxide emissions (SNCR), and a flue gas dust treatment system (mechanical dust separator and bag filter) [54]. The bioheating plant burns approximately 50,000 Mg of wood chips annually, with a dominant share of Scots pine (*Pinus sylvestris* L.) and Norway spruce (*Picea abies* L.) [48] obtained from forests located in the Warmian–Masurian Voivodeship. These wood chips are forest felling residues, primarily mechanically shredded branches and unusable “above-ground” tree parts. A bioheating plant generally produces two types of waste, defined according to Polish [55] and UE law [56] as 10 01 01—slag, bottom ash, and boiler dust; and 10 01 03—fly ash from peat and untreated wood. For the purposes of the conducted research, three waste fractions were separated during bioheating plant operation: B1—grate residue; B2—dust from the dust collector; and B3—boiler dust (Figure 1b–d). In normal operation of a bioheating plant, fractions B1 and B2 are combined and constitute waste designated with code 10 01 03, and fraction B3 is designated as waste with code 10 01 01. The separation of fractions was performed in order to determine the chemical composition of individual waste materials that can be separated during the operation of the bioheating plant.

Samples were collected as weekly aggregate samples during the heating season (2023–2024), over a period of 13 weeks, from 4 February to 4 May 2024 (Table 1). B1 and B2 samples were collected on each of these dates, while B3 samples were collected only in the first 8 weeks of the study, from 4 February to 30 March. In the following weeks, due to technical reasons, sampling of B3 was discontinued.

### 2.2. Analytical Methods

Samples of the obtained waste were dried in an electric dryer at 60 °C, which allowed for the assessment of the dry matter content. The samples were then ground in a porcelain mortar to grind and homogenize the composition. The prepared material was subjected to chemical analyses, which determined: reaction (pH); electrolytic conductivity (EC); content of the following general forms—carbon (TC), nitrogen (TN), phosphorus (P), potassium (K), magnesium (Mg), calcium (Ca), and sodium (Na); and content of general forms of heavy metals, including iron (Fe), manganese (Mn), zinc (Zn), copper (Cu), lead (Pb), cadmium (Cd), chromium (Cr), cobalt (Co), and nickel (Ni). Sample preparation and chemical analyses are presented in Table 2. All analyses and determinations were performed in triplicate.

Contents of heavy metals were determined using standards (Fe 16596, Mn 63534, Zn 188227, Cu 38996, Cd 51994, Pb 16595, Ni 42242, Cr 02733, and Co 119785.0100) (Fluka Company, Charlotte, NC, USA). The obtained results were validated based on the reference material CRM012-100G (Trace Metals—Fly Ash 2, SIGMA-ALDRICH Chemie GmbH, Schnelldorf, Germany).

### 2.3. Statistical Methods

The results obtained were presented as mean content from all sampling dates, as well as the minimum and maximum content for each element. The statistical analysis included the coefficient of variation (CV) and Pearson’s correlation coefficient (*r*). The coefficient of variation allowed for the assessment of the scale of variation in individual characteristics [62,63] based on the entire study period. A CV value less than 20 (CV < 20) indicates low variation in results, a CV value between 20 and 30 (20 < CV < 30) indicates moderate variation, a CV value between 30 and 50 (30 < CV < 50) indicates high variation, and greater than 50 (CV > 50) indicates very high variation [63]. The Pearson coefficient was used to (1) indicate the direction of changes in the content of individual elements with sampling date, (2) indicate the interdependence of the studied characteristics in each material, and (3) indicate the interdependencies of the characteristics in the compared materials. It should be noted that the comparison of materials B1 and B2 was based on 39 variables. Comparisons of B1 with B3 and B2 with B3 were based on 24 variables, due to the smaller number of B3 samples obtained (Table 1). Microsoft Excel^®^ (version 2026) (Microsoft, Redmond, WA, USA) was used for calculations [64], and statistical tables were used to assess the significance of the Pearson coefficient [65].

## 3. Results

### 3.1. General Characteristics of Waste Obtained After Burning Wood Biomass (B1, B2, B3)

Waste samples collected from the Kortowo-Bio bioheating plant were characterized by varied chemical composition, which depended on the waste fraction (Table 3). Taking into account the average content of all distinguished dates for individual sampling sites, B1 samples (residue from the grate) were characterized by the highest average EC value (9.955 mS cm^−1^ DM); the highest content of most of the determined macroelements, including TN (3.091 g), P (8.913 g), K (47.08 g), Mg (27.78 g), Ca (76.51 g), and Na (2.574 g kg^−1^ DM); and heavy metals, including Mn (4254.1 mg), Zn (721.3 mg), Cu (47.59 mg), Pb (46.83 mg), Cd (4.161 mg), Cr (45.08 mg), Co (23.53 mg) and Ni 20.26 (mg kg^−1^ DM) (Table 3). Typically, significantly lower contents of the above-mentioned components were recorded in B2 samples (dust from the dust collector) and the lowest in B3 samples (dust from the boiler) (Table 3). In B2 samples, the values of the above-mentioned elements were from 14 to 72% lower than in B1 samples, while in B3 samples, these differences ranged from 18 to 86%, depending on the component. The highest differences in content were observed for K (49–58%), Ca (50–48%), Zn (72–81%), Cu (50–70%), and Cd (53–73%), and the lowest for TN (18–28%), P (21–38%), Na (15–28%), and Co (16–18%). The exceptions were the average contents of DM and Fe, and pH, the highest values of which were usually recorded in B2 samples. All samples, regardless of the waste type, were characterized by an alkaline reaction (11.65 ≤ pH_KCl_ ≤ 12.66). The average DM content in B2 samples was 99.21%, in B3 it was 96.26%, and in B1 it was much lower at 72.77%. B2 samples were characterized by the highest Fe content (7012.3 mg kg^−1^ DM), which was 15 to 32% higher than in B1 and B3. The average TC content was similar in all samples, regardless of the waste fraction, ranging from 261.8 g (B1) to 265.6 g kg^−1^ DM (B2). However, due to large differences in TN content between samples, the TC/TN ratio varied significantly, ranging from 88.10 in B1 to 129.2 in B3.

Considering the coefficient of variation (CV), among the analyzed samples, the most stable chemical composition with respect to collection dates was observed in B1 samples, while B2 samples were characterized by higher variability, and B3 samples by the highest (Table 3). In B3 samples, the CV value for most of the tested components (EC, TN, TC/TN, P, K, Mg, Na, Mn, Zn, Pb, Cr, and Ni) was characterized by a high degree of variability (32% ≤ CV ≤ 49%), and for Ca, Cu, and Cd it was very high (52% ≤ CV ≤ 78%). However, among the analyzed components, regardless of the waste fraction, the most diversified results were recorded for Cd (69% ≤ CV ≤ 78%) and Pb (35% ≤ CV ≤ 67%). In turn, the pH value of the tested samples and the content of DM and TC in them were usually the least varied (1% ≤ CV ≤ 20%), regardless of the analyzed waste fraction. 

### 3.2. Variation in Parameters in Relation to the Collection Date

#### 3.2.1. DM, pH, EC, TC, TN and TC/TN Values

**Figure 2:**
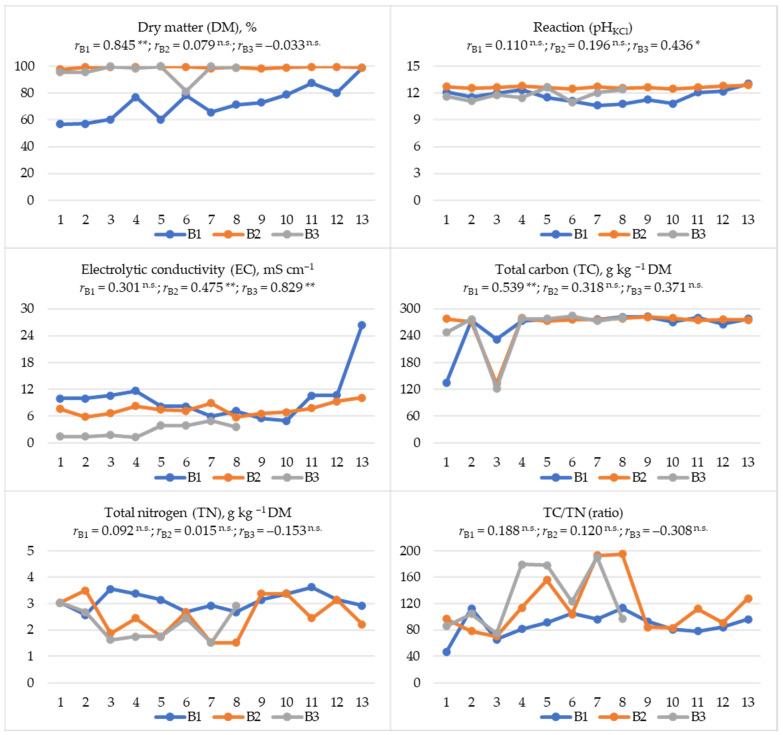
pH and electrolytic conductivity (EC) values, dry matter (DM) content, total carbon (TC) and nitrogen (TN) content, and their ratios (TC/TN) in waste obtained after burning wood biomass. The numbers 1 to 13 indicate the sampling dates; B1—residue from the grate; B2—dust from the dust collector; B3—dust from the boiler; *r*—correlation coefficient; **—significant for *p* ≤ 0.01; *—significant for *p* ≤ 0.05; ^n.s.^—not significant; *n* = 39 for *r*_B1_ and *r*_B2_; *n* = 24 for *r*_B3._

The content of DM, TC and TN, as well as the EC, pH and TC/TN values in combustion waste samples, varied depending on the date of their collection (Figure 2). Based on the correlation coefficient (*r*), it can be concluded that there was a highly significant increase in the DM (*r* = 0.845 **) and TC (*r* = 0.539 **) content in B1 samples, in the EC (*r* = 0.475 **) in B2, and in the pH (*r* = 0.436 *) and EC (*r* = 0.829 **) in B3 (Figure 2). The EC value recorded in B1 samples should also be noted. In the first 10 sampling dates of B1, the EC value ranged from 4.93 to 11.61 mS cm^−1^, showing a decreasing tendency. However, in samples from the last three sampling dates, the EC value increased significantly, reaching a maximum value of 26.43 mS cm^−1^ on the last sampling date. This value was three times higher than the average EC value from the remaining sampling dates. This increase in EC in the last sampling date also contributed to the high CV value mentioned earlier (52%) (Table 3). The least significant changes in relation to the waste collection date (B1, B2, B3) were noted in the TN content and the TC/TN ratio (Figure 2).

In all wastes, positive correlations were observed between EC and pH (0.446 * ≤ *r* ≤ 0.820 **) and negative correlations between TN and TC/TN (−0.799 ** ≤ *r* ≤ −0.730 **) (Tables S1, S4 and S7). Furthermore, in B1 samples, an increase in DM content was accompanied by a significant increase in EC, pH, and TC, and an increase in TC was accompanied by an increase in TC/TN values (Table S1). In turn, the correlation coefficient (*r*) used to indicate the relationships between the contents of the discussed components between the obtained waste fractions revealed that an increase in pH and EC in B1 was accompanied by a highly significant increase in these parameters in B2 (0.595 ** ≤ *r* ≤ 0.737 **), and an increase in TC content in B2 correlated highly significantly with an increase in this component in B3 (Table 4). Negative relationships were noted in the B1:B3 system in the case of DM, EC and TN (−0.910 ** ≤ *r* ≤ −0.437 **).

#### 3.2.2. Macronutrient Contents (P, K, Mg, Ca and Na)

**Figure 3:**
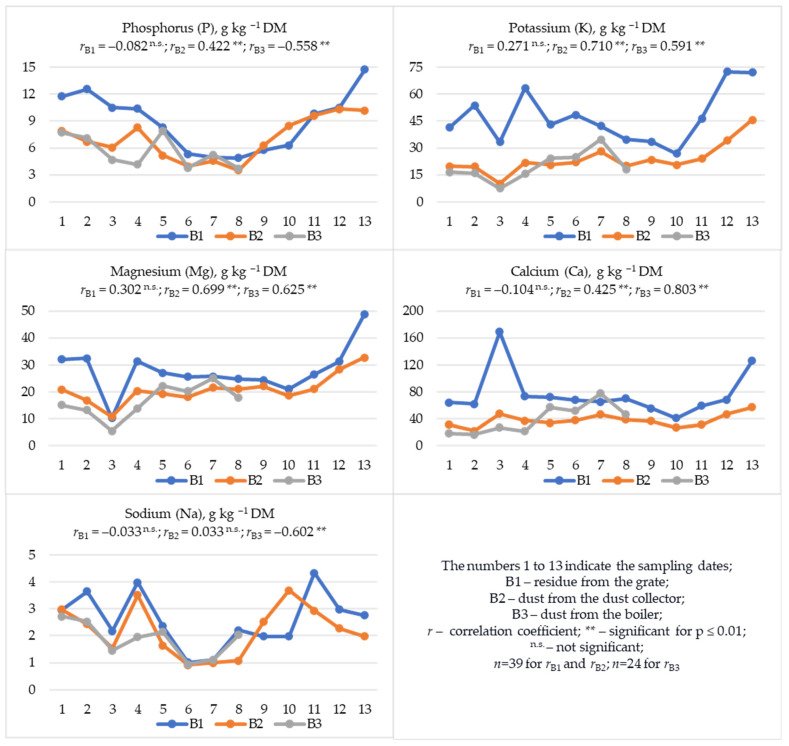
Macronutrient contents (P, K, Mg, Ca, and Na) in waste obtained after burning wood biomass.

Among the macronutrients discussed, regardless of the waste fraction, the dominant content was Ca, K, and Mg (Table 3, Figure 3). Significantly lower P content was observed, and the lowest Na content was found. Of the analyzed samples, fraction B1 was richest in these macronutrients. Despite this, statistical analysis in B1 samples did not reveal a relationship between the content of these macronutrients and the sampling date. However, analyzing the graphs (Figure 3), it can be concluded that the content of P, K, Mg, Ca, and Na in B1 was usually highest in the first and last few sampling dates. The highest results for P (14.75 g) and Mg (48.78 g) were recorded in the last sampling date, and for K in the last two sampling dates (72.47 and 72.14 g kg^−1^ DM, respectively). However, the highest Ca content (168.87 g kg^−1^ DM) was recorded on the third sampling date and was almost 60% higher than the average Ca content in samples from the remaining sampling dates. In B2 samples, there was a clear relationship between the sampling date (Figure 3) and the increase in the content of P, K, Mg, and Ca (0.422 ** ≤ *r* ≤ 0.710 **). This dependence was not observed for the content of Na. In B3 samples, on the other hand, with the increase in the sampling date, the content of K, Mg, and Ca increased (0.591 ** ≤ *r* ≤ 0.803 **), while the content of P and Na decreased (−0.602 ** ≤ *r* ≤ −0.558 **) (Figure 3).

In B1 samples, positive and highly significant correlations were observed in the relationships of P with K, Mg, Ca and Na (0.430 ** ≤ *r* ≤ 0.656 **); K with Mg and Na (0.430 ** ≤ *r* ≤ 0.748 **) (Table S1); In B2 samples, in the relationships of P with K, Mg and Na (0.481 ** ≤ *r* ≤ 0.648 **); K with Mg and Ca (0.568 ** ≤ *r* ≤ 0.951 **); and Mg with Ca (*r* = 0.496 **) (Table S4). In B3 samples, the relationships of P with Na (*r* = 0.612 **); K with Mg and Ca (0.861 ** ≤ *r* ≤ 0.951 **); and Mg with Ca (*r* = 0.817 **) (Table S7). Regardless of the type of waste, the increased Ca content was accompanied by a decrease in Na content; however, this relationship was statistically significant only in B2 and B3 samples (−0.638 ** ≤ *r* ≤ −0.462 **) (Tables S4 and S7). Furthermore, in B1 and B2 samples, the increase in macronutrient content generally significantly correlated with the increase in pH and EC (0.480 ** ≤ *r* ≤ 0.864 **) (Tables S1 and S4). In the case of B3 samples, a positive relationship was observed only between EC and the content of K, Mg, and Ca (0.822 ** ≤ *r* ≤ 0.969 **), and a negative correlation was noted in the relationship between EC and Na (*r* = −0.602 **) (Table S7). Moreover, in B1 samples, positive correlations were observed between the dry matter content and the content of TC, K, Mg, Fe, Mn, Cu, Pb, Cd, Co, and Ni in this material (Tables S1 and S2). Such correlations were not observed in B2 and B3 samples (Tables S4, S5, S7 and S8), where a very high DM content was recorded (Table 3).

Comparing the content of macronutrients in individual waste types and their collection dates (Table 5), the most correlated composition in terms of P, K, Mg, Ca, and Na content was observed in B1 and B2 samples (0.622 ** ≤ *r* ≤ 0.821 **). The correlation coefficient (*r*) value indicates that the increased content of the above-mentioned macronutrients in the grate residue (B1) was accompanied by an increased content in filter dust (B2). Less significant correlations were noted in the compositions of B1 and B3, and B2 and B3. The *r* value indicates positive correlations between the content of P, Mg, and Na in the B1:B3 system and K, Mg, Ca, and Na in the B2:B3 system (Table 5).

#### 3.2.3. Heavy Metal Contents (Fe, Mn, Zn, Cu, Pb, Cd, Cr, Co, and Ni)

**Figure 4:**
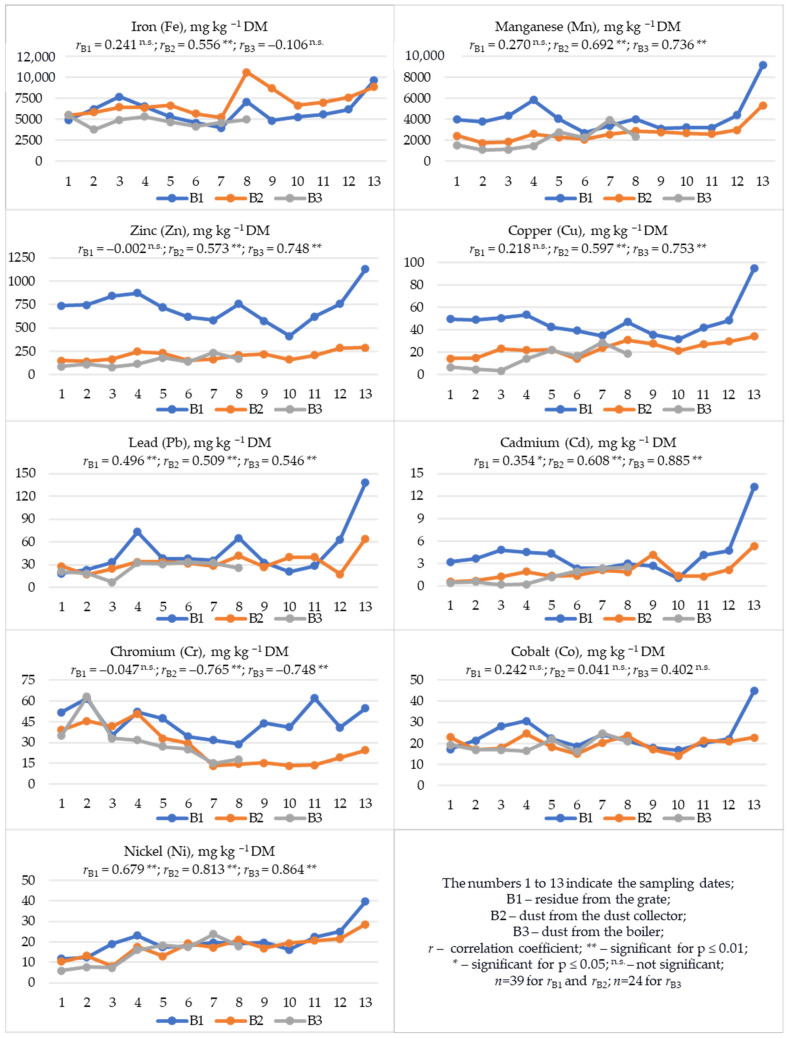
Heavy metal contents (Fe, Mn, Zn, Cu, Pb, Cd, Cr, Co and Ni) in waste obtained after burning wood biomass.

In the waste samples after wood biomass combustion (B1, B2, and B3), regardless of the sampling date, the highest mean ranges of Fe (4759.0–7012.3 mg), Mn (2084.1–4254.1 mg), and Zn (139.7–721.3 mg) contents were observed, and the lowest for Cd (1.175–4.161 mg kg^−1^ DM) (Table 3, Figure 4). Considering the results from individual sampling dates, for most heavy metals (Mn, Zn, Cu, Pb, Cd, Cr, Co), their content was usually the highest in B1 samples (Figure 4). Different results were obtained for Fe and Ni. The Fe content was usually the highest in B2 samples, and the Ni content was comparable in all samples, regardless of the waste type and sampling date. The correlation coefficient (*r*) indicates an increase in metal content in waste with respect to the sampling date (Figure 4). In B1 samples, an increase in the content of Pb, Cd, and Ni was observed (0.354 * ≤ *r* ≤ 0.679 **); in B2 samples, an increase in the content of Fe, Mn, Zn, Cu, Pb, Cd, and Ni (0.509 ** ≤ *r* ≤ 0.813 **); and in B3 samples, an increase in the content of Mn, Zn, Cu, Pb, Cd, and Ni (0.546 ** ≤ *r* ≤ 0.885 **). A different relationship was observed for Cr, whose content in B2 and B3 samples decreased with the elapsed sampling date (−0.765 ** ≤ *r* ≤ −0.748 **). It should be noted that in B1 samples, although the fewest trends were identified, the content of all the heavy metals discussed in this waste increased significantly in the last sampling date (Figure 4).

In B1 samples, the contents of heavy metals, except for Cr, were closely correlated with each other (0.345 * ≤ *r* ≤ 0.946 **) (Table S3). Furthermore, in B1, the contents of all metals were highly significantly and positively correlated with pH (0.562 ** ≤ *r* ≤ 0.778 **), and the vast majority also correlated with EC and the contents of DM, P, K, Mg, and Ca (Table S2). In B2 samples, positive and significant correlations were noted for the contents of Fe, Mn, Zn, Cu, Pb, Cd, and Ni (0.383 * ≤ *r* ≤ 0.790 **) (Table S6). Such relationships were not observed in the composition of B2 for Cr and Co. Moreover, in B2 samples, the content of Mn and Zn was positively correlated with pH, EC, and the content of P, K, Mg, and Ca (0.443 ** ≤ *r* ≤ 0.870 **); Cd with pH, EC, and the content of K, Mg and Ca (0.452 ** ≤ *r* ≤ 0.696 **); and Ni with EC and the content of TC, K, Mg and Ca (0.376 * ≤ *r* ≤ 0.782 **) (Table S5). On the other hand, the content of Pb and Cu showed relationships with some macronutrients, and Co with pH. These correlations were positive. In turn, in B3 samples (Table S9), positive mutual relationships were observed between metals such as Mn, Zn, Cu, Pb, Cd, Co, and Ni (0.517 * ≤ *r* ≤ 0.919 **), and negative relationships between Cr and most metals (−0.701 ** ≤ *r* ≤ −0.451 **). In B3 samples, no significant relationships were observed between Fe and other heavy metals (Table S9) and macroelements (Table S8). All metals detected in B3, except Fe and Cr, showed significant and positive correlations with EC and Ca (0.534 ** ≤ *r* ≤ 0.950 **). Most metals contained in B3 positively correlated with the content of K and Mg (0.561 ** ≤ *r* ≤ 0.911 **). In the composition of B3, no relationship was found between metal contents and pH, except for Co (Table S8).

Comparing the waste composition in terms of the content of the tested metals, the most correlations were noted in the B1:B2 and B1:B3 systems (Table 6). An increase in the content of Fe, Mn, Zn, Pb, Cd, Co, and Ni in B1 resulted in an increase in these components in B2 (0.335 * ≤ *r* ≤ 0.810 **). In the B1:B3 system, most of the observed correlations were negative. Usually, the increase in the content of Mn, Zn, Cu, and Cd in B1 was accompanied by a decrease in the content of these metals in B3 (−0.672 ** ≤ *r* ≤ *−*0.429 *). Only the increase in the content of Pb and Ni in B1 was accompanied by an increase in these metals in B3.

## 4. Discussion

Waste samples from bioheat and power plants, regardless of the tested fraction and sampling date, had an alkaline reaction (11.65 ≤ pH_KCl_ ≤ 12.66) (Table 3, Figure 2), which is characteristic of waste after biomass combustion [23,34,35,38,39,66] or lignite [67]. In the tested materials, regardless of the combustion waste fraction, the dominant content was Ca, K, and Mg, as well as Fe, Mn, and Zn (Table 3). The available literature also indicates a dominant content of Ca [19,31,34,52,53], K [19,52], Zn, and Mn [19], but also P [19,52], S, and Cu [19]. A chemical composition of wood ash similar to the currently presented results was also noted in previous studies on the effect of these wastes on soil properties [38,39]. According to Zając et al. [19], this chemical composition of ashes increases the possibility of using them for fertilizer purposes. According to Du et al. [12], most ashes from woody biomass contain comparable amounts of CaO (up to 46.1%) and SiO_2_ (up to 61.0%), and over 70% of them contain Al_2_O_2_ (<6.75%). This makes them less chemically reactive and more difficult to dissolve even in a strongly alkaline environment [12]. In turn, according to Drljac et al. [29], the composition of wood ash is heterogeneous. The fraction below 100 µm is generally rich in Fe [20], which is confirmed by our research results, indicating the highest content of this metal in the finest B2 fraction. Generally, the high Fe content in combustion waste is justified by the mineral composition of soils in wood-harvesting sites [20]. In turn, samples of fraction B1 (residue from the grate) were characterized by the richest composition in terms of macronutrients (TN, P, K, Mg, Ca, and Na) (Table 3, Figure 2 and Figure 3) and micronutrients valuable for plant nutrition (Mn, Zn, Cu, Co, and Ni) (Table 3, Figure 4). However, it should be noted that fraction B1 was also characterized by the highest salinity level (Table 3), which, with long-term soil application, may cause excessive salt accumulation in the substrate and adversely affect plant growth and development [38,39]. Furthermore, B1 samples also contained the highest amounts of Cd, Pb, and Cr, highly toxic elements whose content in materials used in nature is undesirable. Increased content of toxic metals in wood ashes is also indicated by other authors [31,68], with higher accumulation (Pb and Cd) in the volatile fraction [27,69]. In addition to the significant content of Cd, Pb, Cr, Zn [17,23], Fe, Mn, Cu, and Ni in the ashes [23], the mobility of Co (28.44%), Cr (20.49%), and Pb (16.51%) is also emphasized. The relative enrichment factors (biomass–ash) are much higher for Pb and Ni than for Cd, Cu, Zn, and Cr [20]. As a rule, Zn, Cr, Ni and Pb contained in the ash are associated with manganese, iron, and aluminum oxides; Cu is associated with the residual fraction; and Cd is associated with the soluble fraction, iron, manganese and aluminum oxides and the residual fraction [53]. The leaching process of heavy metals from wood ashes decreases as they age [27]. However, the irregularity of ash grains may favor the release of heavy metals, which may lead to environmental pollution [23]. The release of components contained in ash and their accumulation in plants may depend on meteorological conditions, primarily on the amount of precipitation, which has a direct impact on the solubility of components present in ash [70]. The highest leachability of chlorides, potassium, and sulfates is observed in ash from fluidized bed boilers [4]. Ash from woody biomass is characterized by a high yield of solutions soluble in alkaline water. Components such as Ca, Cl, K, Mg, Na, P, and S occur in significant amounts in water-soluble and bioavailable salts [18]. This could explain the significant correlations obtained in our study between the DM content and the pH and EC values, as well as the contents of TC, K, Mg, Fe, Mn, Cu, Pb, Cd, Co and Ni in fraction B1 (Tables S1 and S2), which was the most moist material (Table 3, Figure 2). The proportions of elements extracted with water from ash and their release over time are very important for predicting the supply of bioavailable macronutrients or elements toxic to crops [18]. This is especially important in soils with increased metal contents, where an increase in the share of available Cd and Pb in their total content is observed after the addition of ash [48].

Environmental hazards may also be posed by high concentrations of alkali metals and chlorine, which may also cause technical problems, including slagging, contamination, and an increased risk of leaching after application to soil [17]. This is influenced by the type of furnace [4,71]. Ash from furnaces with lower efficiency has higher K concentrations, but also higher Cd concentrations. Higher furnace efficiency results in a higher content of the coarser wood ash fraction, which is of lower fertilizer value [3]. Combustion of biomass in a grate furnace allows for obtaining a larger amount of K than from a fluidized bed furnace [72].

According to some [73], all metals except Hg are retained in the ash, and at temperatures below 1000 °C, the metals are evenly distributed in each ash particle. At higher temperatures, they are lost from the outer layers. According to Madea et al. [72], a small amount of K may be released with exhaust gases, but most of this component remains in the ash. Partial (incomplete) combustion usually favors higher P, Ca, and Mg contents in the ash [66]. Other researchers, in turn, indicate that burning wood at too-low temperatures can cause increased Pb and Cd emissions into the atmosphere [20], and long-term storage of ash can cause water and soil pollution [20,29,74]. The type of biomass burned is of great importance in the combustion process: burning wood briquettes usually produces higher Pb and Cd emissions than burning spruce wood [20]; wood residues, especially sawdust, contain high levels of contaminants, e.g., Al [75]; pellets made from wood waste usually contain preservatives [76]; and wood ash is generally characterized by higher Cd and Pb, As, and Hg contents than ashes from the combustion of agricultural residues [68,69]. It should be noted that the origin of the wood also plays a key role in this respect. Burning waste wood usually results in higher heavy metal content in combustion waste [7]. Furthermore, wood from industrial waste and energy crops is characterized by higher Na, K, P, and Cl contents; higher ash content; and high corrosion potential [77].

In the presented studies, variability in the content of most of the analyzed components in combustion waste fractions was observed in relation to the sampling date (Figure 2, Figure 3 and Figure 4). For many elements (TC, P, K, Mg, Ca, Mn, Zn, Cu, Pb, Cd, Ni), a tendency to increase in content was observed with the sampling date (Figure 2, Figure 3 and Figure 4). According to some researchers [53], this is the result of the quality of the feedstock used in the bioheating plant. In the case of metallic elements, this usually indicates wear of part of the technological line because of the corrosive effect of woody biomass [22,49,50,78], which is intensified due to the content of S and Cl in the biomass [22,78]. Also, the presence of K, P and Na in the biomass in the form of unfavorable compounds (chlorides, sulfates, carbonates, oxalates, phosphates, nitrates) may also pose serious technological and environmental challenges during biomass processing [79].

Among the analyzed combustion waste fractions, the highest content of macronutrients (TN, P, K, Mg, Ca, Na) and valuable micronutrients (Mn, Zn, Cu, Co, and Ni) as well as a high content of TC were found in fraction B1 (Table 3), making this material very interesting for environmental applications, including fertilization. Considering the average content of the analyzed components (Table 3), 1 ton of this waste fraction would contribute to the soil 261.8 kg of TC, 76.51 kg of Ca, 47.08 kg of K, 27.78 kg of Mg, 8.913 kg of P, 3.091 kg of TN, and 2.574 kg of Na, as well as significant amounts of Fe (5.991 kg), Mn (4.254 kg), and Zn (0.721 kg). The results are all the more interesting given that fraction B1 is also a final waste requiring rational management. In normal bioheating plant operation, fraction B1 is mixed with fraction B2 and referred to as fly ash. Because fraction B2 was less rich in most of the components tested, there is no risk of over-enrichment of the target waste with undesirable components. Therefore, mixing of dust from the bag filter with grate ash, as exemplified by this unit, seems justified. It should be noted that both fractions (B1 and B2) were also characterized by lower variability in the content of individual components than fraction B3 (Table 3), which, from an environmental application point of view, is the desired result. Furthermore, the increase in most parameters (pH, EC, P, K, Mg, Ca, Na, Fe, Mn, Zn, Pb, Cd, Co, and Ni) in fraction B1 was accompanied by their increase in fraction B2 (Table 3, Table 4 and Table 5). According to Vasiliev et al. [18], the use of such products for the production of fertilizer materials is particularly promising, including macronutrient, micronutrient, and multinutrient fertilizers, as well as liming agents, soil improvers, and plant biostimulants. The production of calcium fertilizers with the addition of biomass combustion waste using a molasses-based binder is also promising [80]. Supplementing N content is also important in the production of such materials, due to its low content in combustion waste [39]. The production of fertilizers from waste products would contribute to the sustainable development of fertilizer production and, thus, to the recycling of industrial by-products [80]. A very important aspect when choosing a natural method of waste management is the content of heavy metals, of which Pb, Cd, Cr, and Ni deserve special attention. These, along with Hg and As, are the most frequently limited elements in fertilizers or soil conditioners in the applicable legal regulations in this area [81,82,83]. The most stringent limits can be found in Regulation (EU) 2019/1009 of the European Parliament and of the Council [83], which limits the content of heavy metals in inorganic soil improvers to 1.5 mg Cd, 100 mg Ni, 120 mg Pb, 2 mg Cr(VI), 300 mg Cu, and 800 mg Zn kg^−1^ DM. Considering the recorded Cd content in the tested fraction B1 (Figure 4), in most cases it would not meet these requirements. The Pb content in fraction B1 sampled on the last date was also higher than the limits for this product established in the cited regulation. We cannot refer to the Cr content, because in our studies, we determined its total content without releasing Cr(VI), the limit of which is specified in the aforementioned regulation [83]. The Cu content was within the specified limits, but the Zn content in B1 samples from three dates (3, 4, and 13) exceeded the established limits for this element. Looking at the basis of Polish laws and regulations [81,82], the indications are less stringent. The lowest content limit in organic and organo-mineral plant growth enhancers was set at 5 mg Cd, 60 mg Ni, 140 mg Pb, and 10 mg Cr per kg^−1^ of DM, and in mineral-based plant growth enhancers, other than lime fertilizer and lime fertilizer containing magnesium, at 50 mg Cd and 140 mg Pb per kg^−1^ of DM. Ni and Cr content are not limited here. Therefore, Polish standards would be more favorable to the results obtained and the possibilities of environmentally sustainable management of this waste.

To ensure the environmentally sound management of ash, pretreatment methods should be developed [7], including acid leaching, thermal treatment, or mixing with inert materials, to reduce the mobility of contaminants and increase the availability of nutrients [17]. Before deciding to use wood ash as a secondary raw material, it is necessary to conduct leaching tests to determine which micronutrients it contains and which of them can be released into the environment [29]. Ash from bioheating plants that use unprocessed-wood biofuels may be suitable for use as fertilizer if specific requirements regarding contaminant and nutrient contents are met [84]. Comprehensive assessments of long-term leaching of components from ash are necessary to estimate the potential impact of their use on the environment [5]. Furthermore, long-term field studies are also needed to assess the impact of annual ash application on soil properties, element bioavailability, and microbial dynamics [17,19]. To avoid health risks, caution should be exercised when using ash from wood contaminated with metals [40], as this may promote the accumulation of these elements in plants [41]. According to some, the use of higher ash doses may lead to excessive alkalization and salinization of soils. These aspects should therefore be closely monitored, especially in the case of long-term use of this waste, to avoid serious environmental problems [41]. Furthermore, persistent organic pollutants (POPs) should be considered, as they tend to be released into exhaust gases and may pose additional environmental risks [31].

## 5. Conclusions

In all furnace waste fractions considered, the dominant macronutrients were Ca (38.05–76.51 g), K (19.74–47.08 g), and Mg (16.62–27.78 g), with significantly lower P (5.546–8.913 g) and Na (1.857–2.574 g kg^−1^ DM). Among heavy metals, the highest content was recorded for Fe (4759.0–7012.3 mg), Mn (2084.1–4254.1 mg), and Zn (139.7–721.3 mg), and the lowest for Cd (1.175–4.161 mg kg^−1^ DM). All combustion waste samples, regardless of fraction, were characterized by an alkaline reaction (11.65 ≤ pH_KCl_ ≤ 12.66) and similar TC content (261.8–265.6 g kg^−1^ DM). Among the waste fractions tested, B1 samples had the highest mean EC value (9.955 mS cm^−1^ DM), the highest content of most macronutrients (TN, P, K, Mg, Ca, Na), and valuable micronutrients (Mn, Zn, Cu, Cr, Co, Ni), but also undesirable heavy metals (Pb, Cd). The exception was the mean content of DM and Fe, as well as pH, the highest values of which were recorded in B2 samples.

Among the waste fractions, the most diverse composition was observed in B3 samples. Among the analyzed components, regardless of the waste fraction type, the most variable results were observed for Cd and Pb. This variability was related to sampling dates. In fraction B1, the contents of P, K, Mg, Ca, and Na were typically highest in the first and last few terms, while the contents of Mn, Zn, Cu, Pb, Cd, Co, Cr, and EC increased significantly on the last sampling date. Furthermore, increases in most parameters (pH, EC, P, K, Mg, Ca, Na, Fe, Mn, Zn, Pb, Cd, Co, and Ni) in fraction B1 were accompanied by increases in fraction B2. Fewer correlations were noted between B1 and B3 and between B2 and B3.

The obtained results clearly indicate that fraction B1 (residue from the grate), due to its high content of macro- and microelements, is the most valuable material in the context of environmental management. However, it requires constant monitoring of composition for heavy metal content, primarily Cd and Pb, and salinity.

## Figures and Tables

**Table 1:** Type of samples and their collection date.

Sample No.	Sample Collection Date	Sample Type		
		B1	B2	B3
1	4–10 February 2024	x	x	x
2	11–17 February 2024	x	x	x
3	18–24 February 2024	x	x	x
4	25 February–2 March 2024	x	x	x
5	3–9 March 2024	x	x	x
6	10–16 March 2024	x	x	x
7	17–23 March 2024	x	x	x
8	24–30 March 2024	x	x	x
9	31 March–6 April 2024	x	x	–
10	7–13 April 2024	x	x	–
11	14–20 April 2024	x	x	–
12	21–27 April 2024	x	x	–
13	28 April–4 May 2024	x	x	–

B1—Residue from the grate; B2—dust from the dust collector; B3—dust from the boiler; x—sample taken; – no sample taken.

**Table 2:** Analytical methods used in the research.

Elements	Methods and Apparatus
Reaction (pH_KCl_)	Potentiometric method; KCl solution with a concentration of 1 mol dm^−3^; pH 538 laboratory pH meter, WTW electrode (WTW, Wrocław, Poland) [57].
Electric conductivity (EC)	Conductivity method; HANNA HI8733 conductivity meter (Hanna Instruments, Leighton Buzzard, UK) [57].
Total carbon (TC)	TOC Analyser (Shimadzu Corporation, Kyoto, Japan), SSM-5000A adapter (Solid Sample Module, Shimadzu Corporation, Kyoto, Japan).
Total nitrogen (TN)	Kjeldahl distillation method [58]; concentrated sulfuric acid (VI) with hydrogen peroxide; wet digestion—Speed Digester K-439 digestion furnace (BÜCHI Labor-technik AG, Flawil, Switzerland); scrubber K-415 vapor absorber (BÜCHI Labor-technik AG, Flawil, Switzerland); distillation—K-355 steam still (BÜCHI Labor-technik AG, Flawil, Switzerland).
Total forms: P, K, Mg, Ca and Na	P—vanadium–molybdenum method [59]; Mg—atomic absorption spectrometry (AAS); and K, Ca, and Na—flame atomic emission spectroscopy (FAES) [59]; AA240FS Fast Sequential Atomic Absorption Spectrometer (Varian Inc., Mulgrave, Australia). Contents of these elements were determined in the same mineralized samples used for TN.
Total forms: Fe, Mn, Zn, Cu, Pb, Cd, Co, Cr, and Ni	ASA method using the AA240FS Fast Sequential Atomic Absorption Spectrometer (Varian Inc., Mulgrave, Australia). Contents of these elements were determined after wet mineralization of the samples using MARS 6 microwave oven (CEM Corporation, Matthews, NC, USA) in MARS Xpress teflon vessels according to the US-EPA 3051 methodology [60,61]. Acid solutions (65% HNO_3_ and 38% HCl) were used in a ratio of 3:1 (*v*/*v*).

**Table 3:** Selected physicochemical properties of waste obtained after burning wood biomass (average values for all terms).

Elements	Sample Type					
	B1		B2		B3	
	Mean (CV%)	Min–Max	Mean (CV%)	Min–Max	Mean (CV%)	Min–Max
Dry matter (DM) (%)	72.77 (17)	56.97–98.84	99.21 (1)	97.86–99.86	96.26 (6)	81.46–99.85
Reaction (pH_KCl_)	11.65 (6)	10.61–13.03	12.66 (1)	12.47–12.87	11.76 (5)	10.99–12.64
EC (mS cm^−1^ DM)	9.955 (52)	4.930–26.43	7.556 (17)	5.773–10.12	2.767 (49)	1.433–4.927
TC (g kg^−1^ DM)	261.8 (15)	133.9–282.7	265.6 (15)	132.2–282.1	254.9 (20)	122.2–284.3
TN (g kg^−1^ DM)	3.091 (18)	2.567–3.617	2.531 (31)	1.517–3.500	2.216 (34)	1.517–3.033
Ratio TC/TN	88.10 (27)	46.77–113.4	115.9 (40)	70.7–195.4	129.2 (45)	75.0–190.5
P (g kg^−1^ DM)	8.913 (36)	4.905–14.75	7.009 (34)	3.538–10.35	5.546 (32)	3.708–7.910
K (g kg^−1^ DM)	47.08 (30)	26.86–72.47	23.85 (34)	10.09–45.73	19.74 (39)	7.56–34.81
Mg (g kg^−1^ DM)	27.78 (30)	10.31–48.78	20.89 (24)	10.75–32.76	16.62 (35)	5.50–24.99
Ca (g kg^−1^ DM)	76.51 (42)	40.76–168.9	38.05 (24)	22.21–56.93	39.56 (52)	16.77–77.35
Na (g kg^−1^ DM)	2.574 (38)	1.015–4.326	2.191 (41)	0.917–3.695	1.857 (32)	0.939–2.716
Fe (mg kg^−1^ DM)	5990.5 (24)	3989.0–9602.9	7012.3 (22)	5240.1–10,624.8	4759.0 (15)	3809.2–5525.0
Mn (mg kg^−1^ DM)	4254.1 (38)	2732.7–9153.2	2695.1 (31)	1759.0–5337.0	2084.1 (44)	1096.2–3941.4
Zn (mg kg^−1^ DM)	721.3 (23)	413.3–1130.8	202.4 (27)	142.2–290.2	139.7 (37)	79.63–233.7
Cu (mg kg^−1^ DM)	47.59 (32)	31.70–94.77	23.58 (34)	14.30–34.30	14.40 (63)	3.500–28.57
Pb (mg kg^−1^ DM)	46.83 (67)	18.53–138.2	32.94 (36)	17.10–64.20	25.16 (35)	7.000–32.53
Cd (mg kg^−1^ DM)	4.161 (69)	1.050–13.22	1.959 (70)	0.567–5.350	1.175 (78)	0.167–2.484
Cr (mg kg^−1^ DM)	45.08 (25)	28.98–61.95	27.19 (49)	13.27–50.73	31.07 (46)	15.07–62.97
Co (mg kg^−1^ DM)	23.53 (32)	16.77–44.87	19.72 (20)	14.30–24.67	19.19 (19)	16.10–24.87
Ni (mg kg^−1^ DM)	20.26 (34)	11.77–39.57	17.41 (31)	8.033–28.47	14.35 (43)	6.033–23.77

B1—Residue from the grate; B2—dust from the dust collector; B3—dust from the boiler; CV—coefficient of variation%; EC—electrolytic conductivity; TC—total carbon; TN—total nitrogen.

**Table 4:** Correlation coefficient (*r*) between DM, pH, EC, TC, TN, TC/TN in the analyzed materials (B1, B2 and B3).

Elements	Type of Samples and Their Relationships		
	B1:B2	B1:B3	B2:B3
DM	0.182 ^n.s.^	−0.437 **	−0.114 ^n.s.^
pH	0.737 **	−0.280 ^n.s.^	0.232 ^n.s.^
EC	0.595 **	−0.910 **	0.265 ^n.s.^
TC	0.215 ^n.s.^	0.364 *	0.971 **
TN	−0.073 ^n.s.^	−0.453 **	0.351 ^n.s.^
TC/TN	0.157 ^n.s.^	0.033 ^n.s.^	0.418 ^n.s.^

B1—Residue from the grate; B2—dust from the dust collector; B3—dust from the boiler; DM—dry matter; EC—electrolytic conductivity; TC—total carbon; TN—total nitrogen; TC/TN—ratio; *r*—correlation coefficient; **—significant for *p* ≤ 0.01; *—significant for *p* ≤ 0.05; ^n.s.^—not significant; *n* = 39 for *r*_B1_ and *r*_B2_; *n* = 24 for *r*_B3._

**Table 5:** Correlation coefficient (*r*) between macroelements (P, K, Mg, Ca, and Na) in the analyzed materials (B1, B2, and B3).

Elements	Types of Sample and Their Relationships		
	B1:B2	B1:B3	B2:B3
P	0.622 **	0.551 **	0.304 ^n.s.^
K	0.706 **	0.046 ^n.s.^	0.880 **
Mg	0.821 **	0.419 **	0.755 **
Ca	0.641 **	−0.208 ^n.s.^	0.516 *
Na	0.631 **	0.771 **	0.665 **

B1—Residue from the grate; B2—dust from the dust collector; B3—dust from the boiler; *r*—correlation coefficient; **—significant for *p* ≤ 0.01; *—significant for *p* ≤ 0.05; ^n.s.^—not significant; *n* = 39 for *r*_B1_ and *r*_B2_; *n* = 24 for *r*_B3_.

**Table 6:** Correlation coefficient (*r*) between heavy metals (Fe, Mn, Zn, Cu, Pb, Cd, Cr, Co and Ni) in the analyzed materials (B1, B2 and B3).

Elements	Types of Sample and Their Relationships		
	B1:B2	B1:B3	B2:B3
Fe	0.527 **	0.123 ^n.s.^	0.133 ^n.s.^
Mn	0.810 **	−0.429 *	0.478 *
Zn	0.499 **	−0.672 **	0.150 ^n.s.^
Cu	0.302 ^n.s.^	−0.654 **	0.187 ^n.s.^
Pb	0.664 **	0.412 *	0.513 *
Cd	0.612 **	−0.629 **	0.446 *
Cr	0.316 ^n.s.^	0.795 **	0.712 **
Co	0.335 *	−0.096 ^n.s.^	0.159 ^n.s.^
Ni	0.694 **	0.576 **	0.637 **

B1—Residue from the grate; B2—dust from the dust collector; B3—dust from the boiler; *r*—correlation coefficient; **—significant for *p* ≤ 0.01; *—significant for *p* ≤ 0.05; ^n.s.^—not significant; *n* = 39 for *r*_B1_ and *r*_B2_; *n* = 24 for *r*_B3._

## Data Availability

The original contributions presented in this study are included in the article/Supplementary Material. Further inquiries can be directed to the corresponding author.

## Supplementary Materials

The following supporting information can be downloaded at https://www.mdpi.com/article/10.3390/ma19071295/s1: Table S1. The value of the correlation coefficient (*r*) between macronutrients in fraction B1; Table S2. The value of the correlation coefficient (*r*) between macronutrients and heavy metals in fraction B1; Table S3. The value of the correlation coefficient (*r*) between in fraction B1; Table S4. The value of the correlation coefficient (*r*) between macronutrients in fraction B2; Table S5. The value of the correlation coefficient (*r*) between macronutrients and heavy metals in fraction B2; Table S6. The value of the correlation coefficient (*r*) between in fraction B2; Table S7. The value of the correlation coefficient (*r*) between macronutrients in fraction B3; Table S8. The value of the correlation coefficient (*r*) between macronutrients and heavy metals in fraction B3; Table S9. The value of the correlation coefficient (*r*) between in fraction B3.
