# Supplementary material for: Variability of Properties of Wood Biomass Combustion Waste During the Heating Season in the Context of Their Environmental Use
Source: Materials (Basel). 2026 Mar 25;19(7):1295. doi: 10.3390/ma19071295 (PMC13073439; doi:10.3390/ma19071295)
Supplement: Supplementary file 1 [file materials-19-01295-s001.zip › materials-4209385-supplementary.pdf]

materials

MDPI

Article

Variability of properties of wood biomass combustion waste during the heating season in the context of their environmental use

Elżbieta Rolka *, Anna Skorwider-Namiotko and Radosław Szostek

Department of Agricultural and Environmental Chemistry, Faculty of Agriculture and Forestry, University of Warmia and Mazury in Olsztyn, Łódzki 4 Sq., 10-727 Olsztyn, Poland; elzbieta.rolka@uwm.edu.pl (E.R.); anna.namiotko@uwm.edu.pl (A.S.-N.); radoslaw.szostek@uwm.edu.pl (R.S.)

* Correspondence: elzbieta.rolka@uwm.edu.pl (E.R.)

Table S1. The value of the correlation coefficient (*r*) between macronutrients in fraction B1.

Elements	DM	pH	EC	TC	TN	TC/TN	P	K	Mg	Ca
pH	0.376*									
EC	0.556**	0.820**								
TC	0.453**	–0.245 n.s.	–0.010 n.s.							
TN	0.111 n.s.	0.172 n.s.	–0.004 n.s.	–0.051 n.s.						
TC/TN	0.125 n.s.	–0.295 n.s.	–0.030 n.s.	0.593**	–0.799**					
P	0.132 n.s.	0.864**	0.750**	–0.323 n.s.	0.016 n.s.	–0.187 n.s.				
K	0.467**	0.700**	0.694**	0.149 n.s.	–0.084 n.s.	0.109 n.s.	0.617**			
Mg	0.472**	0.558**	0.738**	0.033 n.s.	–0.221 n.s.	0.150 n.s.	0.552**	0.748**		
Ca	0.016 n.s.	0.480**	0.548**	–0.135 n.s.	0.116 n.s.	–0.147 n.s.	0.430**	0.140 n.s.	–0.094 n.s.	
Na	0.149 n.s.	0.640**	0.336*	–0.091 n.s.	0.149 n.s.	–0.153 n.s.	0.656**	0.430**	0.335*	–0.044 n.s.

B1—Residue from the grate; DM—dry matter; EC—electrolytic conductivity; TC—total carbon; TN—total nitrogen; **—significant for $p \leq 0.01$; *—significant for $p \leq 0.05$; n.s.—not significant; *n*=39.

Table S2. The value of the correlation coefficient (*r*) between macronutrients and heavy metals in fraction B1.

Elements	DM	pH	EC	TC	TN	TC/TN	P	K	Mg	Ca	Na
Fe	0.400*	0.639**	0.800**	0.095 n.s.	0.026 n.s.	0.057 n.s.	0.612**	0.429**	0.379*	0.699**	0.322 n.s.
Mn	0.490**	0.740**	0.920**	0.032 n.s.	–0.021 n.s.	0.007 n.s.	0.664**	0.645**	0.709**	0.527**	0.286 n.s.
Zn	0.240 n.s.	0.778**	0.867**	–0.092 n.s.	–0.056 n.s.	–0.008 n.s.	0.715**	0.656**	0.578**	0.675**	0.349*
Cu	0.454**	0.773**	0.963**	–0.051 n.s.	–0.087 n.s.	0.025 n.s.	0.747**	0.649**	0.721**	0.575**	0.303 n.s.
Pb	0.659**	0.562**	0.824**	0.293 n.s.	–0.080 n.s.	0.194 n.s.	0.386*	0.664**	0.684**	0.409*	0.116 n.s.
Cd	0.531**	0.772**	0.958**	0.077 n.s.	–0.005 n.s.	0.040 n.s.	0.677**	0.647**	0.689**	0.586**	0.271 n.s.
Cr	0.151 n.s.	0.582**	0.409*	–0.096 n.s.	0.058 n.s.	–0.083 n.s.	0.662**	0.381*	0.494**	–0.109 n.s.	0.769**
Co	0.457**	0.624**	0.844**	0.180 n.s.	0.048 n.s.	0.053 n.s.	0.539**	0.574**	0.544**	0.647**	0.158 n.s.
Ni	0.783**	0.570**	0.784**	0.362*	0.110 n.s.	0.078 n.s.	0.343*	0.616**	0.564**	0.409*	0.104 n.s.

B1—Residue from the grate; DM—dry matter; EC—electrolytic conductivity; TC—total carbon; TN—total nitrogen; **—significant for $p \leq 0.01$; **—significant for $p \leq 0.05$; n.s.—not significant; *n*=39.

Table S3. The value of the correlation coefficient (*r*) between elements in fraction B1.

Elements	Fe	Mn	Zn	Cu	Pb	Cd	Cr	Co
Mn	0.831**							
Zn	0.841**	0.886**						
Cu	0.855**	0.946**	0.921**					
Pb	0.760**	0.901**	0.787**	0.844**				
Cd	0.805**	0.916**	0.860**	0.935**	0.848**			
Cr	0.178 n.s.	0.282 n.s.	0.256 n.s.	0.333*	0.068 n.s.	0.345*		
Co	0.786**	0.907**	0.834**	0.856**	0.851**	0.877**	0.157 n.s.	
Ni	0.663**	0.789**	0.632**	0.742**	0.868**	0.820**	0.129 n.s.	0.784**

B1—Residue from the grate; **—significant for p ≤ 0.01; **—significant for p ≤ 0.05; n.s.—not significant; *n*=39.

Table S4. The value of the correlation coefficient (*r*) between macronutrients in fraction B2.

Elements	DM	pH	EC	TC	TN	TC/TN	P	K	Mg	Ca
pH	–0.123 n.s.									
EC	–0.016 n.s.	0.728**								
TC	–0.241 n.s.	0.002 n.s.	0.192 n.s.							
TN	–0.214 n.s.	–0.148 n.s.	–0.106 n.s.	0.242 n.s.						
TC/TN	–0.027 n.s.	0.087 n.s.	0.117 n.s.	0.296 n.s.	–0.776**					
P	0.066 n.s.	0.514**	0.519**	0.119 n.s.	0.365*	–0.371*				
K	–0.078 n.s.	0.587**	0.780**	0.475**	0.009 n.s.	0.188 n.s.	0.481**			
Mg	–0.195 n.s.	0.626**	0.715**	0.579**	0.052 n.s.	0.215 n.s.	0.493**	0.951**		
Ca	0.009 n.s.	0.632**	0.649**	–0.281 n.s.	–0.452**	0.243 n.s.	0.097 n.s.	0.568**	0.496**	
Na	–0.102 n.s.	0.124 n.s.	0.030 n.s.	0.235 n.s.	0.560**	–0.457**	0.648**	–0.033 n.s.	0.057 n.s.	–0.462**

B2—Dust from the dust collector; DM—dry matter; EC—electrolytic conductivity; TC—total carbon; TN—total nitrogen; **—significant for p ≤ 0.01; *—significant for p ≤ 0.05; n.s.—not significant; *n*=39.

Table S5. The value of the correlation coefficient (*r*) between macronutrients and heavy metals in fraction B2.

Elements	DM	pH	EC	TC	TN	TC/TN	P	K	Mg	Ca	Na
Fe	–0.036 n.s.	0.127 n.s.	–0.076 n.s.	0.129 n.s.	–0.148 n.s.	0.225 n.s.	0.033 n.s.	0.303 n.s.	0.436**	0.298 n.s.	–0.131 n.s.
Mn	–0.154 n.s.	0.577**	0.647**	0.288 n.s.	–0.106 n.s.	0.193 n.s.	0.462**	0.870**	0.862**	0.626**	0.035 n.s.
Zn	0.209 n.s.	0.633**	0.545**	0.191 n.s.	–0.120 n.s.	0.120 n.s.	0.443**	0.623**	0.681**	0.507**	0.080 n.s.
Cu	0.018 n.s.	0.402*	0.297 n.s.	0.030 n.s.	–0.279 n.s.	0.286 n.s.	0.213 n.s.	0.444**	0.480**	0.480**	–0.085 n.s.
Pb	–0.011 n.s.	0.176 n.s.	0.347*	0.206 n.s.	–0.311 n.s.	0.310 n.s.	0.193 n.s.	0.512**	0.486**	0.361 n.s.	0.014 n.s.
Cd	–0.192 n.s.	0.474**	0.452**	0.155 n.s.	–0.029 n.s.	0.080 n.s.	0.247 n.s.	0.696**	0.672**	0.607**	–0.092 n.s.
Cr	0.256 n.s.	0.170 n.s.	–0.118 n.s.	–0.328*	0.073 n.s.	–0.303 n.s.	0.007 n.s.	–0.351*	–0.385*	–0.168 n.s.	0.157 n.s.
Co	–0.154 n.s.	0.586**	0.372*	0.160 n.s.	–0.251 n.s.	0.270 n.s.	0.209 n.s.	0.267 n.s.	0.385*	0.275 n.s.	0.069 n.s.
Ni	0.083 n.s.	0.215 n.s.	0.473**	0.493**	–0.035 n.s.	0.227 n.s.	0.332*	0.782**	0.757**	0.376*	–0.009 n.s.

B2—Dust from the dust collector; DM—dry matter; EC—electrolytic conductivity; TC—total carbon; TN—total nitrogen; **—significant for p ≤ 0.01; *—significant for p ≤ 0.05; n.s.—not significant; *n*=39.

https://doi.org/10.3390/xxxxx

Table S6. The value of the correlation coefficient (*r*) between elements in fraction B2.

Elements	Fe	Mn	Zn	Cu	Pb	Cd	Cr	Co
Mn	0.543**							
Zn	0.519**	0.646**						
Cu	0.553**	0.540**	0.558**					
Pb	0.448**	0.776**	0.383*	0.422**				
Cd	0.549**	0.790**	0.598**	0.506**	0.515**			
Cr	–0.433**	–0.331*	–0.153 n.s.	–0.409*	–0.289 n.s.	–0.315 n.s.		
Co	0.266 n.s.	0.350*	0.355*	0.270 n.s.	0.250 n.s.	0.086 n.s.	0.059 n.s.	
Ni	0.507**	0.754**	0.575**	0.478**	0.647**	0.587**	–0.522**	0.177 n.s.

B2—Dust from the dust collector; **—significant for p ≤ 0.01; *—significant for p ≤ 0.05; n.s.—not significant; *n*=39.

Table S7. The value of the correlation coefficient (*r*) between macronutrients in fraction B3.

Elements	DM	pH	EC	TC	TN	TC/TN	P	K	Mg	Ca
pH	0.671**									
EC	–0.115 n.s.	0.446*								
TC	–0.272 n.s.	–0.021 n.s.	0.351 n.s.							
TN	–0.280 n.s.	–0.200 n.s.	–0.212 n.s.	0.248 n.s.						
TC/TN	0.130 n.s.	0.218 n.s.	0.361 n.s.	0.382 n.s.	–0.730**					
P	0.215 n.s.	0.167 n.s.	–0.159 n.s.	0.076 n.s.	0.101 n.s.	–0.021 n.s.				
K	–0.169 n.s.	0.206 n.s.	0.834**	0.621**	–0.146 n.s.	0.497*	0.046 n.s.			
Mg	–0.168 n.s.	0.322 n.s.	0.822**	0.749**	–0.040 n.s.	0.492*	0.091 n.s.	0.951**		
Ca	–0.021 n.s.	0.482*	0.969**	0.299 n.s.	–0.298 n.s.	0.411 n.s.	–0.150 n.s.	0.861**	0.817**	
Na	0.350 n.s.	0.097 n.s.	–0.602**	0.139 n.s.	0.409 n.s.	–0.210 n.s.	0.612**	–0.424*	–0.259 n.s.	–0.638**

B3—Dust from the boiler; DM—dry matter; EC—electrolytic conductivity; TC—total carbon; TN—total nitrogen; **—significant for p ≤ 0.01; *—significant for p ≤ 0.05; n.s.—not significant; *n*=24.

Table S8. The value of the correlation coefficient (*r*) between macronutrients and heavy metals in fraction B3.

Elements	DM	pH	EC	TC	TN	TC/TN	P	K	Mg	Ca	Na
Fe	0.311 n.s.	0.283 n.s.	–0.258 n.s.	–0.187 n.s.	0.122 n.s.	–0.006 n.s.	–0.115 n.s.	–0.222 n.s.	–0.161 n.s.	–0.166 n.s.	0.165 n.s.
Mn	0.069 n.s.	0.504 n.s.	0.911**	0.415 n.s.	–0.274 n.s.	0.510*	–0.073 n.s.	0.911**	0.882**	0.950**	–0.478*
Zn	0.127 n.s.	0.498*	0.831**	0.493*	–0.283 n.s.	0.544**	–0.114 n.s.	0.842**	0.830**	0.868**	–0.370 n.s.
Cu	0.061 n.s.	0.436*	0.806**	0.506*	–0.209 n.s.	0.490*	–0.226 n.s.	0.801**	0.821**	0.834**	–0.423*
Pb	–0.265 n.s.	0.083 n.s.	0.534**	0.814**	–0.009 n.s.	0.481*	–0.127 n.s.	0.750**	0.820**	0.560**	–0.266 n.s.
Cd	–0.189 n.s.	0.330 n.s.	0.873**	0.483*	0.034 n.s.	0.239 n.s.	–0.357 n.s.	0.717**	0.755**	0.810**	–0.477*
Cr	–0.048 n.s.	–0.520*	–0.692**	–0.083 n.s.	0.206 n.s.	–0.239 n.s.	0.433*	–0.498*	–0.519*	–0.726**	0.553**
Co	0.365 n.s.	0.592**	0.586**	0.211 n.s.	–0.151 n.s.	0.299 n.s.	0.130 n.s.	0.561**	0.567**	0.593**	–0.043 n.s.
Ni	0.007 n.s.	0.389 n.s.	0.817**	0.521*	–0.322 n.s.	0.589**	–0.391 n.s.	0.786**	0.791**	0.850**	–0.559**

B3—Dust from the boiler; DM—dry matter; EC—electrolytic conductivity; TC—total carbon; TN—total nitrogen; **—significant for p ≤ 0.01; *—significant for p ≤ 0.05; n.s.—not significant; *n*=24.

https://doi.org/10.3390/xxxxx

Table S9. The value of the correlation coefficient (*r*) between elements in fraction B3.

Elements	Fe	Mn	Zn	Cu	Pb	Cd	Cr	Co
Mn	–0.045 n.s.							
Zn	–0.115 n.s.	0.912**						
Cu	–0.004 n.s.	0.887**	0.820**					
Pb	–0.042 n.s.	0.617**	0.596**	0.683**				
Cd	–0.194 n.s.	0.796**	0.779**	0.736**	0.517*			
Cr	–0.282 n.s.	–0.701**	–0.615**	–0.680**	–0.451*	–0.640**		
Co	0.112 n.s.	0.664**	0.720**	0.592**	0.209 n.s.	0.481*	–0.535*	
Ni	–0.050 n.s.	0.865**	0.847**	0.919**	0.753**	0.763**	–0.681**	0.446*

B3—Dust from the boiler; **—significant for $p \leq 0.01$; *—significant for $p \leq 0.05$; n.s.—not significant; n=24.

Disclaimer/Publisher's Note: The statements, opinions and data contained in all publications are solely those of the individual author(s) and contributor(s) and not of MDPI and/or the editor(s). MDPI and/or the editor(s) disclaim responsibility for any injury to people or property resulting from any ideas, methods, instructions or products referred to in the content.

https://doi.org/10.3390/xxxxx
